# Gelatin Nanoparticles-HPMC Hybrid System for Effective Ocular Topical Administration of Antihypertensive Agents

**DOI:** 10.3390/pharmaceutics12040306

**Published:** 2020-03-28

**Authors:** Sergio Esteban-Pérez, Vanessa Andrés-Guerrero, José Javier López-Cano, Irene Molina-Martínez, Rocio Herrero-Vanrell, Irene Bravo-Osuna

**Affiliations:** 1 Innovation, Therapy and Pharmaceutical Development in Ophthalmology (InnOftal) Research Group, UCM 920415, Department of Pharmaceutics and Food Technology, Faculty of Pharmacy, Complutense University of Madrid, Plaza Ramón y Cajal s/n, 28040 Madrid, Spain; seresteb@ucm.es (S.E.-P.); vandres@ucm.es (V.A.-G.); josejavl@ucm.es (J.J.L.-C.); iremm@ucm.es (I.M.-M.); rociohv@ucm.es (R.H.-V.); 2 Sanitary Research Institute of the San Carlos Clinical Hospital (IdISSC) San Carlos Clinical Hospital, Calle Profesor Martín Lagos, s/n, 28040 Madrid, Spain; 3 Ocular Pathology National Net (OFTARED) of the Institute of Health Carlos III, Calle Profesor Martín Lagos, s/n, 28040 Madrid, Spain

**Keywords:** gelatin nanoparticles, glaucoma, HPMC, timolol maleate, topical ocular drug delivery, nanotechnology

## Abstract

The increment in ocular drug bioavailability after topical administration is one of the main challenges in pharmaceutical technology. For several years, different strategies based on nanotechnology, hydrogels or implants have been evaluated. Nowadays, the tolerance of ophthalmic preparations has become a critical issue and it is essential to the use of well tolerated excipients. In the present work, we have explored the potential of gelatin nanoparticles (GNPs) loaded with timolol maleate (TM), a beta-adrenergic blocker widely used in the clinic for glaucoma treatment and a hybrid system of TM-GNPs included in a hydroxypropyl methylcellulose (HPMC) viscous solution. The TM- loaded nanoparticles (mean particle size of 193 ± 20 nm and drug loading of 0.291 ± 0.019 mg TM/mg GNPs) were well tolerated both *in vitro* (human corneal cells) and *in vivo.* The *in vivo* efficacy studies performed in normotensive rabbits demonstrated that these gelatin nanoparticles were able to achieve the same hypotensive effect as a marketed formulation (0.5% TM) containing a 5-fold lower concentration of the drug. When comparing commercial and TM-GNPs formulations with the same TM dose, nanoparticles generated an increased efficacy with a significant (*p* < 0.05) reduction of intraocular pressure (IOP) (from 21% to 30%) and an augmentation of 1.7-fold in the area under the curve (AUC)_(0–12h)_. On the other hand, the combination of timolol-loaded nanoparticles (TM 0.1%) and the viscous polymer HPMC 0.3%, statistically improved the IOP reduction up to 30% (4.65 mmHg) accompanied by a faster time of maximum effect (t_max_ = 1 h). Furthermore, the hypotensive effect was extended for four additional hours, reaching a pharmacological activity that lasted 12 h after a single instillation of this combination, and leading to an AUC_(0–12h)_ 2.5-fold higher than the one observed for the marketed formulation. According to the data presented in this work, the use of hybrid systems that combine well tolerated gelatin nanoparticles and a viscous agent could be a promising alternative in the management of high intraocular pressure in glaucoma.

## 1. Introduction

Glaucoma is considered as a group of ocular chronic disorders characterized by a distinctive neuropathy that leads to a progressive asymptomatic visual field loss. One of the main risk factors in glaucoma development is the high intraocular pressure (IOP) [1,2]. Generally, the maintained increase of IOP is triggered by the accumulation of aqueous humour in the anterior chamber of the eye due to a distortion between its production and its outflow through the trabecular meshwork. This high pressure is transmitted to the posterior segment of the eye and to the retina and optic nerve head promoting the progressive death of the retinal ganglion cells (RGCs) and thus, inducing the loss of vision [3].

Currently, lowering of IOP is the only therapeutic measure for glaucoma management. Glaucoma treatments consist of IOP modulation by either reducing the aqueous humour production or enhancing its outflow. Among the main therapeutic agents, non-selective β- antagonists such as timolol maleate (TM), betaxolol or carteolol are widely employed as first-line therapy because of their efficacy at reducing IOP [4]. These drugs reduce aqueous humour by inhibition of AMPc synthesis via antagonization of the β-adrenergic receptors present in the ciliary body cells [5].

Topical ocular administration is the chosen route for IOP management. Eye drops can be straightforwardly instilled on the ocular surface in a comfortable way for patients. However low ocular bioavailability, typically lower than 5%, is generally achieved for classical eye drops formulations [6,7]. This is due to the presence of ocular protective barriers and precorneal tear clearance that reduce the drug residence time on the ocular surface. Furthermore, most active compounds present poor corneal penetration [8,9]. This is the reason why repeated administrations and high drug concentrations are required in order to obtain a therapeutic effect [10]. Unfortunately, in many cases repeated administrations lead to low patient compliance and thus, therapeutic failure happens [11]. Moreover, part of the lost formulation can be drained through the nasolacrimal channel, reaching the bloodstream and provoking undesirable systemic effects. For example, TM has been related to different systemic adverse reactions when eye drops are administered in a chronical way. Adverse effects such as cardiac effects, bronchospasm or syncope among others have typically been associated with chronical treatments with this active compound [9,12,13].

The development of new formulations enhancing drug permeation, bioavailability and safety profile after ocular instillation has become one of the most important challenges in pharmaceutical technology in recent years [14,15,16]. Several novel drug delivery systems have been developed like nanoparticles, microparticles, microemulsions, hydrogels or contact lenses among others, to achieve more effective therapies [17,18,19,20,21]. However, it is mandatory to keep always in mind that all excipients used must be highly compatible with ocular tissues [22]. Most efforts that have been made focused on increasing the retention time of formulations, and the contained drug, on the ocular surface as an initial step for the passive transport of the active compounds through the cornea [8,20,23]. An effective method to retain the formulation on the ocular surface is the addition of polymers able to increase the formulation viscosity and/or its mucoadhesion, such as gelatin, chitosan, hydroxypropyl methylcellulose (HPMC), gellan, carboxy methylcellulose (CMC), hyaluronic acid, polyacrylate acids, and xanthan gums among others. This strategy partially avoids the ocular surface clearance produced by the tearing and blinking reflex [24,25,26,27,28]. In fact, several authors have successfully explored the use of the polysaccharide HPMC in this context [29,30,31]. Furthermore, HPMC is widely employed as comfort agent for dry eye disease (DED) due to its pseudoplastic behaviour and high tolerance [32]. Because of the chronic nature of glaucoma and the need of repeated instillations, many patients suffer also DED over time, due to the continuous contact with preservatives and even with some drugs such as timolol maleate [33]. Studies from our research group have demonstrated that the inclusion of HPMC in the formulation increases its *in vitro* and *in vivo* tolerance in experimental animals [24].

As mentioned before, another interesting approach to increase the drug retention time on the ocular surface and, hence, its ocular bioavailability, is the use of nanoparticles [22,34,35]. Due to their nanometric range and large surface area, they seem to have an enhanced capacity to be entrapped over the precorneal film and even to permeate the ocular surface epithelium [36]. In addition, nanoparticles can protect the drug from degradation and modulate its release in a controlled manner [37,38]. Several biopolymers have been evaluated to prepare nanoparticles for ocular instillation, such as polylactic-co-glycolic acid [39,40], chitosan [37,41] or hyaluronic acid [42,43]. In this sense, gelatin is a widely employed material due to its biocompatible and biodegradable characteristics [44,45]. It has mucoadhesive properties that can prolong nanoparticles precorneal residence time [46,47]. The first experiments with gelatin nanoparticles for topical administration originate from 1989, in which authors employed pilocarpine for miotic purposes [48]. Later, some researchers developed nanoparticles for topical administration that contained pilocarpine and hydrocortisone [49]. More recently, other authors have explored the use of gelatin nanoparticles with other active agents such as timolol maleate [50] or moxifloxacin [51]. Nowadays, the rise in genetic therapies has also shown that gelatin nanosystems are safe and efficient vectors for gene delivery in ophthalmology [52].

The aim of the present work is to evaluate the combination of two technological strategies to increase the ocular bioavailability of timolol maleate (TM), an ocular hypotensive drug widely used in the clinic for glaucoma treatment. To that end, we have explored the potential of a hybrid system composed by gelatin nanoparticles loaded with TM, included in a hydroxypropyl methylcellulose viscous solution. Timolol-loaded gelatin nanoparticles (GNPs) were prepared by an ethanol-water solvent displacement method that avoids the use of hazardous components during the process. Formulations were physicochemically characterized. In order to mimic ocular surface conditions, the *in vitro* release tests were performed with proteases or metalloproteinase-2 (MMP-2) in the release media, which are enzymes typically present in the eye of glaucomatous patients [53,54,55]. These results were compared with conventional release studies performed with plain PBS solutions. After *in vitro* (human corneal epithelium cells) and *in vivo* (rabbits) tolerance studies, the hypotensive efficacy of the novel formulations with different TM concentrations was evaluated in rabbits and compared with the administration of a timolol maleate marketed formulation under the same conditions.

## 2. Materials and Methods

### 2.1. Materials

Gelatin from bovine skin type B (gel strength 225 g Bloom), protease from *Streptomyces griseus*, NaOH, MMP-2 human recombinant, glyoxal solution 40% and glycine were purchased from Sigma-Aldrich (St. Louis, MO, USA). TM and HPMC were obtained from Fagron (Barcelona, Spain). Absolute ethanol, trifluoroacetic acid and acetonitrile were acquired from Panreac (Madrid, Spain). Dialysis membrane with a molecular weight cut-off 3500 g/mol was provided by Medicel Membranes (London, UK). Timabak 0.5% (Thea Laboratories; Madrid, Spain) was purchased in a local pharmacy store. Ultrapure milli-Q water was used in all the studies. All other chemicals were reagent grade and used as received.

### 2.2. TM HPLC Determination

The TM high-performance-liquid-chromatography (HPLC) determination was performed as described by Boiero et al. [56] with slight modifications. The HPLC system was composed by a separation module Waters Alliance 2695 and a Waters photodiode array 2996 (Barcelona, Spain). The wavelength was set at 297 nm. Mobile phase was composed by 0.05% (v/v) trifluoroacetic acid in water and 0.05% (v/v) trifluoroacetic acid in acetonitrile (40:60) at a flow rate of 1 mL/min. The analytical column was Hypersil silica column (250 × 4 mm 5 µm particle size). Data acquisition and processing were performed by Empower 3 software (Waters, Barcelona, Spain).

### 2.3. Elaboration of Gelatin Nanoparticles (GNPs) and Eye Drops Formulations

GNP formulations were developed employing a modified ethanol-water solvent displacement method as described by Amiji and co-workers [57]. Briefly, 200 mg type B gelatin (Bloom 225) was dissolved in 20 mL of ultrapure water at 37 °C, then TM (200 mg) was dissolved in the so-formed gelatin solution and pH was adjusted to 7.0 using a solution of NaOH 0.2M. Nanoparticles were created by the ethanol-induced desolvation process. Briefly, the gelatinous solution was added dropwise to ~65 mL of absolute ethanol under permanent stirring (600 rpm). The resulting nanoparticulate structures were crosslinked with 1mL of 4% glyoxal. After 2 min of contact, unreacted glyoxal groups were blocked by 5 mL of glycine 1mM. Later, the resulting suspension was dialyzed overnight against 5L of ultrapure water to warrant the elimination of any soluble compound. Finally, the nanoparticle suspension was freeze-dried to obtain a free-flowing product.

Eye drops formulations were prepared by a simple suspension of nanoparticles in PBS isotonized with NaCl (pH 7.4). No aggregation was observed in any of the formulations prepared. In the case of the hybrid system proposed, timolol maleate loaded nanoparticles were suspended in HPMC 0.3% solution in the previous mentioned media.

**Scheme 1:**
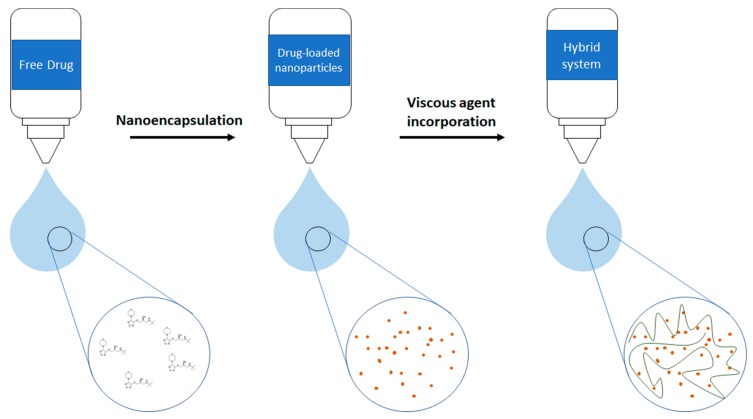
General structure of timolol maleate (TM) and the gelatin nanoparticles–hydroxypropyl methylcellulose (HPMC) hybrid system.

Formulations have been coded as “GNP” for blank gelatin nanoparticles and “TM-GNP” for timolol-loaded gelatin nanoparticle formulations as follows: TM-GNP XX (TM YY %). Where XX is the concentration (mg/mL) of nanoparticles employed and YY is % TM (w/w) for each formulation. Scheme 1 represents a general structure of TM and the gelatin nanoparticles-HPMC hybrid system proposed in this work.

### 2.4. Measurement of Mean Particle Size, Particle Size Distribution, Zeta Potential and Polydispersity Index of the TM-GNP

The mean particle size, particle size distribution and polydispersity index of nanoparticles were measured after the resuspension of the freeze-dried product in ultrapure water and Z-potential after PBS dispersion, using the dynamic light scattering technique with the instrument Malvern Zetasizer (Malvern, Worcestershire, UK). Determinations were assessed by triplicate. PDI value was determined using the following equation:

$$\mathrm{PDI}={\left(\frac{\sigma }{D}\right)}^{2}$$

where *D* is the mean diameter of the GNPs and the *σ* is the standard deviation [58].

### 2.5. Transmission Electron Microscopy

GNP and TM-GNP were observed using JEOL TEM 1010 transmission electron microscopy. For this purpose, 1 mg/mL of resuspended nanoparticulate formulations was drop-casted onto a copper grid (Electron Microscopic Sciences; Aname; Rioja, Spain) and, after 30 s of incubation, the copper grid with the nanoparticles was dropped on a 2% uranyl acetate (Merck; Madrid, Spain) solution as a contrast imaging agent before observation.

### 2.6. Determination of TM Encapsulation Efficiency

In order to determine the amount of hypotensive drug encapsulated in TM-GNPs (n = 13), 2 mg of nanoparticles were suspended in a solution of protease (0.2 mg/mL) in PBS and incubated at 37 °C for 20 min to promote the nanoparticle digestion. Then, the clear solution was filtered through a 0.22 micrometers filter and thus, analyzed by HPLC according to Section 2.2.

### 2.7. In Vitro Release Study

The release media employed for *in vitro* release assays was prepared with PBS isotonized with NaCl. The pH measurement of PBS was performed with a pH-meter (model 230, Mettler, Barcelona, Spain) equipped with a microelectrode (InLab, Mettler, Barcelona, Spain) resulting in a value of 7.4, similar to the precorneal film pH [59].

An amount of TM-GNP to reach 5 mg/mL of TM (equivalent concentration as manufactured formulation) was reconstituted in 1 mL of PBS with or without protease (60 ng/mL) or MMP-2 (60 ng/mL) (n = 4) and incorporated to a Float-A-Lyzer device (molecular weight cut-off 5,000 g/mol). The device was then submerged in a tube with 12 mL of PBS. The study was performed in a shaking bath at 33 °C and 100 rpm. At pre-determined times (0.5, 3, 6, 12, 24, 48, 72 and 96 h), 12 mL of release media were taken and used for the quantification of TM, then, the same volume of fresh media was added to continue the study.

The drug loading value obtained in the determination of encapsulation efficiency assay previously described in Section 2.6 was considered as the 100% of drug that can potentially be released from the nanoparticles.

### 2.8. In Vitro Tolerance Studies

Human corneal cells (HCEpiC) (Innoprot; Bizkaia, Spain) were grown in the corneal epithelial cell medium supplemented with Human Corneal Growth Factors (HCGF) and 50 IU/mL penicillin, 50 µg/mL streptomycin, containing 5 mM glucose (Sigma Aldrich). Cells were incubated at 37 °C in a 5% carbon dioxide atmosphere under 95% humidity and experiments were performed under passages 5–10. Cells were grown as monolayers and were employed after becoming confluent. Twenty-four-well plates and cells at a 2 × 10^4^ cells/well concentration were seeded for the *in vitro* tolerance study. The MTT ((3-(4,5-Dimethylthiazol-2-yl)-2,5-Diphenyltetrazolium Bromide) (Sigma Aldrich) cytotoxicity assay was used [15,60]. MTT technique is widely employed for assessing toxicity in novel developed systems [14,15]. Cells were exposed to gelatin (30 mg/mL), GNP (10 mg/mL and 30 mg/mL) and TM-GNP (10 mg/mL, 20 mg/mL and 30 mg/mL) at different exposure times (1 h, 4 h and 24 h). Then MTT (5mg/mL) reactive was added and incubated for 4 h. After the incubation period, dimethyl sulfoxide was added to solubilize the formazan salts. The absorbance was read at 570 nm with background subtraction at 690 nm.

Benzalkonium chloride (BAK) 0.005% was used as a positive cytotoxicity control [61]. Untreated cells were used as negative control. The percentage of viable cells was determined in relation to control cells. To that end, the optical density of control cells was divided by the optical density of the treated cells and multiplied per 100 and thus, the tolerance value was obtained.

### 2.9. Animals

Healthy normotensive male New Zealand rabbits with an average weight of 3 kg (Granja San Bernardo, Navarra, Spain) were kept at an animal house of the Complutense University of Madrid. Animals were housed in separated cages with food, water ad libitum and were maintained in a controlled environment at 25 °C with a 12-h on/off light cycle. All procedures herein complied with the Association for Research in Vision and Ophthalmology (ARVO) Statement for the Use of Animals in Ophthalmic and Vision Research and were also in accordance with the European Communities Council Directive (2010/63/UE of the European Parliament and of the Council of 22 September 2010 on the protection of animals used for scientific purposes) and approved by the Complutense University of Madrid and Autonomous Community of Madrid (PROEX 316/16 January 25 2017).

### 2.10. In Vivo Acute Tolerance Tests

*In vivo* tolerance studies were performed in rabbits (n = 6). According to the protocol, 25 µl of a GNP suspension (30 mg/mL) was administered in the right eye (OD) of the rabbit and 25 µl of an isotonic saline solution in the left eye (OI) every 30 min for six hours. The macroscopic evaluation was assessed before the beginning the study and at 3, 6 and 24 h after instillation. The scoring system for the macroscopic evaluation was performed according to the modified protocol established by Enriquez et al., used by our research group in previous studies [62,63,64]. The clinical signs observed were corneal opacity, conjunctival alterations, discharge, eyelid swelling and animal discomfort (as shown by intense blinking), and were sorted from 0 to 2 (Table 1).

### 2.11. In Vivo Efficacy Study

Animals received a single dose (25 µl) of the different tested formulations. TM-GNP concentration in each formulation was adjusted to an equivalent concentration of TM (0.5%, 0.25% or 0.1%) that resulted 16 mg/mL, 8 mg/mL or 3 mg/mL, respectively. Non-loaded GNP were tested at 16 mg/mL (n = 20 per group) as control. A marketed formulation (TM 0.5%) was employed as a reference. In a second step of the study, in light of the results obtained, TM-GNP (3 mg/mL; TM 0.1%) was suspended in HPMC 0.3% to determine the efficacy of the nanoparticles in a viscous solution.

To study the time course of the effect of the different formulations, two IOP measurements were taken before any compound was administered (30 min and just before the instillation) to determine basal IOP. Then, measurements were taken at pre-scheduled times (1, 2, 3, 4, 5, 6, 7, 8, 12 and 24 h after instillation). All measurements were performed with an Icare Tonovet rebound tonometer (Vantaa; Finland). This technique allows the acquisition of IOP values without the administration of topical anaesthesia. The parameters analyzed were the area under the curve (AUC) from 0–12 h, the maximum % IOP reduction, hypotensive duration of the effect and time where the maximum effect is reached (tmax).

### 2.12. Statistical Analysis

All data are presented as the mean ± Standard Error (SE). Statistical differences for *in vitro* tolerance and hypotensive determination at *in vivo* studies between two mean values were assessed by two-tailed students’ *t*-test. For comparative purposes of inferiority, equality or superiority, ANOVA between the developed formulations and the manufactured reference formulation have been performed. The software used to perform the mentioned tests were Statgraphics Centurion 18 (Virginia; USA) and Graphpad Prism 6 (San Diego, CA, USA).

## 3. Results

### 3.1. Timolol Maleate Loaded Gelatin Nanoparticles Characterization

**Figure 1:**
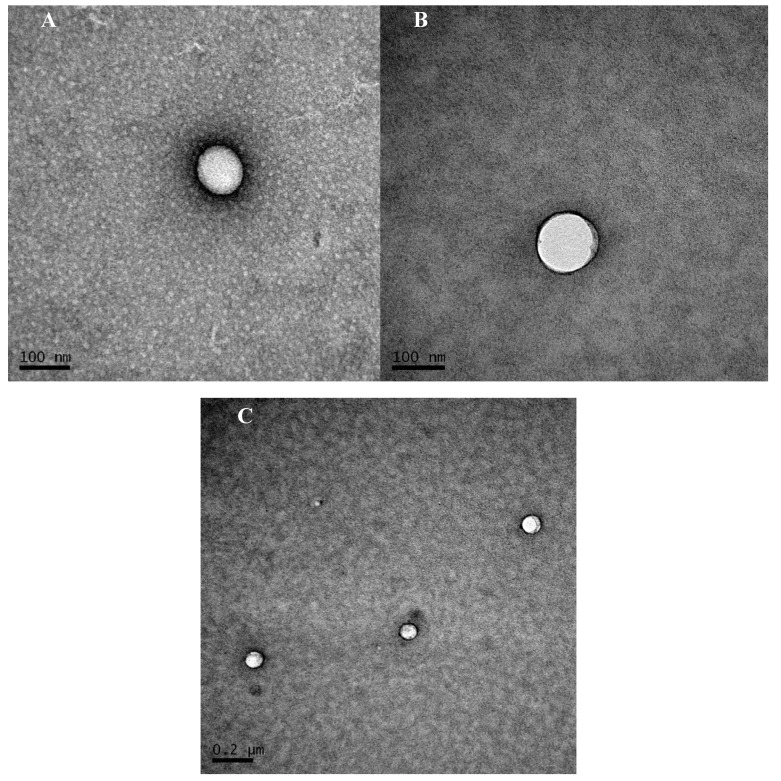
TEM images of the nanoparticles developed. Image (**A**) shows gelatin nanoparticles (GNP), whereas images (**B**,**C**) corresponds with TM-GNP.

TM-GNP presented a round shape according to TEM images (Figure 1), with a mean size of 193.2 ± 20.7 nm and 0.011 PDI and a neutral Z-potential (-0.684 ± 4.48 mV). TM-GNP presented a drug loading of 0.291 ± 0.019 mg TM/mg GNPs (58.7 ± 3.8%).

### 3.2. In Vitro Release Studies

**Figure 2:**
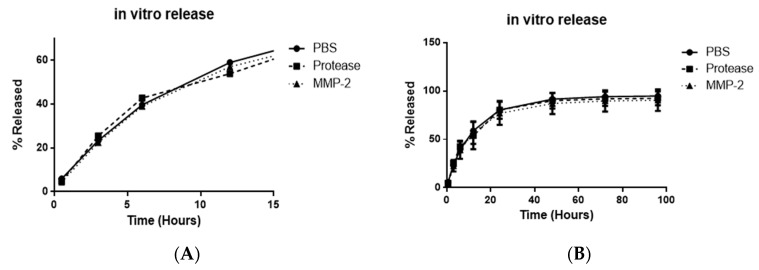
(**A**) Release points at 0.5, 3 and 6 h, deviation marks have been removed in order to permit its appreciation (**B**) Cumulative *in vitro* release (percentage) of TM for 96 h (5 days) with three different release media PBS, PBS with protease (60 ng/mL) and PBS with MMP-2 (60 ng/mL). Each experiment was carried out by quadruplicate.

Nanoparticles released the 5.90 ± 1.04% of the loading drug in the first 30 min of release assay in PBS. After that, 80.46 ± 1.44% of the total timolol maleate was delivered at the end of the first day of study. The remaining content was sustained and released during the next 24 h at a rate of 11.14% TM/day (from day 1 to day 2 of the release test) and 1.64% TM/day from day 2 to day 4 of the release test (Figure 2). When the released media was modified by the inclusion of proteases or MMP-2 similar values were obtained (see values inserted in Table 2 and Figure 2). According to the similarity factor values, similarity was found in the release profile when protease or metalloproteinase was included (f2 = 89 for PBS versus Protease profiles and f2 = 71 for PBS versus MMP-2 profiles) [65].

### 3.3. In Vitro and in Vivo Tolerance Studies

**Figure 3:**
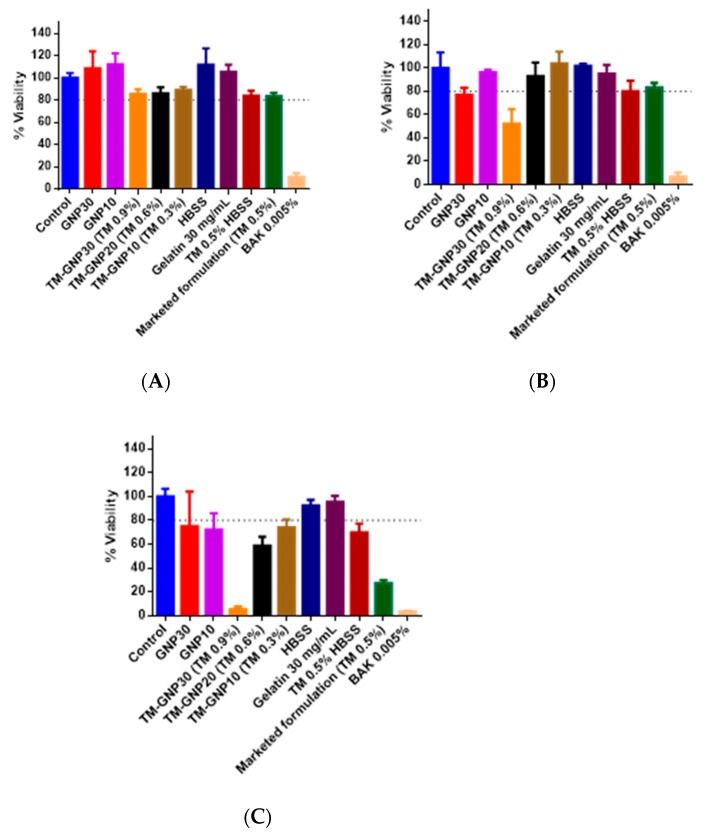
Human corneal cells (HCEpiC) viability at different exposition times (1 h (**A**), 4 h (**B**) and 24 h (**C**)). Negative control: cell culture media; GNP30, GNP20 and GNP10 entail unloaded gelatin nanoparticles at 30, 20 and 10 mg/mL, respectively. TM-GNP30, TM-GNP20 and TM-GNP10 indicate timolol maleate gelatin loaded nanoparticles at 30 mg/mL, 20 mg/mL and 10 mg/mL (equivalent percentage of timolol maleate shown in brackets: 0.9%, 0.6% and 0.3%). HBSS: Hank’s balanced salt solution; gelatin 30 mg/mL dissolved in HBSS (raw material), Timolol maleate 0.5% dissolved in HBSS (raw material), marketed formulation (0.5%) and positive control BAK 0.005%.

According to the MTT studies, unloaded gelatin nanoparticles up to concentrations of 30mg/mL were well tolerated by corneal cells after 1h, 4h and 24h of contact time (cell viability values higher than 80% in all cases). However, when particles were loaded with timolol maleate, a dose-dependent reduction of cell viability was observed after 4h and 24h of exposition. This reduction was also observed for the commercial preparation after 24h of contact. TM-GNP at 20 mg/mL and 10 mg/mL resulted in viability values higher than 80% after exposures of 1 h and 4 h with a decrease at 24 h. It is worthy to mention that the marketed formulation was less tolerated by corneal cells than 20 mg/mL TM-GNP, which contained a higher concentration of the active compound (Figure 3).

An acute *in vivo* tolerance study was performed with non-loaded nanoparticles (30 mg/mL). Right before the first instillation, rabbits presented no clinical signs and ocular structures were as usual. No corneal discomfort or eyelid inflammation was observed at 3, 6 and 24 h after a single instillation. Some of the treated eyes presented a minimal depot around the rabbit eye for 24 h after the beginning of the study. The *in vivo* efficacy studies were performed with the formulation that generated the best results in the *in vitro* cytotoxicity studies and *in vivo* tolerance studies, and therefore resulted in the best tolerance.

### 3.4. In Vivo Efficacy Studies

**Figure 4:**
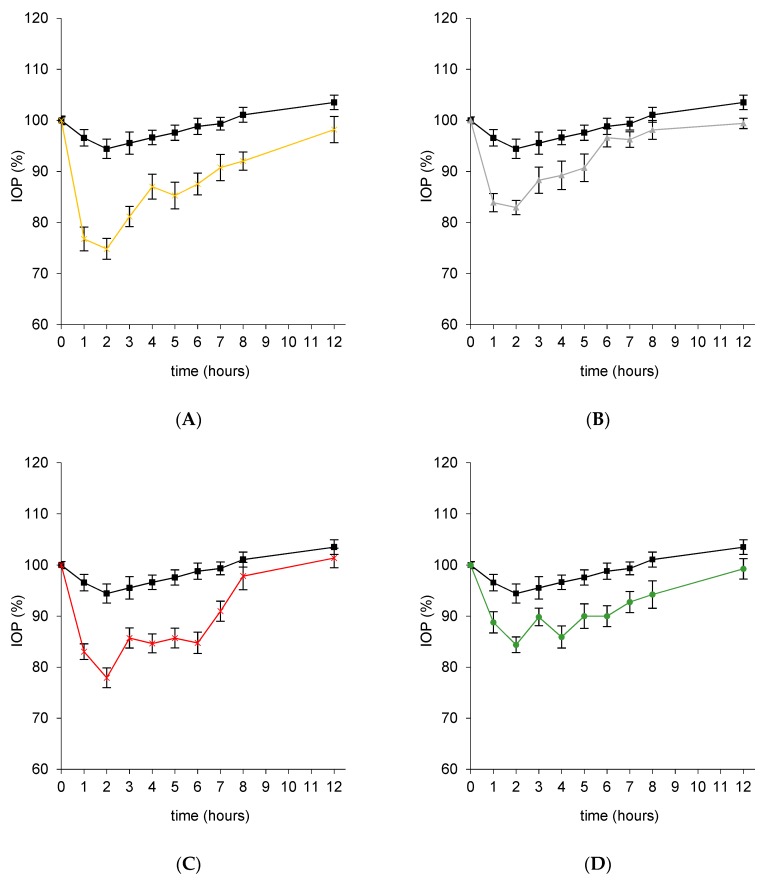
Intraocular pressure (IOP) reduction generated by a single instillation of the following formulations: (**A**) TM-GNP16 (TM 0.5%), (**B**) marketed formulation (TM 0.5%, used as a reference), (**C**) TMGNP8 (TM 0.25%) and (**D**) TMGNP3 (TM 0.1%). In black, IOP reduction curve of unloaded gelatin nanoparticles used as control.

IOP measurements were performed at pre-set times from 0 to 24 h after a single instillation of the different formulations. The empty nanocarrier (non-loaded gelatin nanoparticles (GNP) were tested as control, showing small IOP variations resulting in no significant differences to basal values (p > 0.05). For comparison purposes, studies were also performed with a marketed formulation (0.5%) used as reference (Figure 4).

In a first set of experiments different concentrations of timolol-loaded nanoparticles (16 mg/mL, 8 mg/mL, and 3 mg/mL) corresponding to TM concentrations of 0.5%, 0.25% and 0.1%, respectively were tested and compared with the marketed formulation (timolol maleate content of 0.5%) (Figure 4 and Table 3). The loading of TM in gelatin nanoparticles induced an increment in drug efficacy in terms of maximal IOP reduction and also drug bioavailability (expressed as AUC _(0–12)_), when TM was administered at the same dose of the marketed formulation (p-values shown in Table 4).

In fact, it was necessary to reduce nearly five times the concentration of TM-GNP to achieve comparable IOP values with the marketed formulation. On the contrary, the commercial preparation generated a faster onset of action, showing a t_max_ 30 min earlier than TM-GNPs.

**Figure 5:**
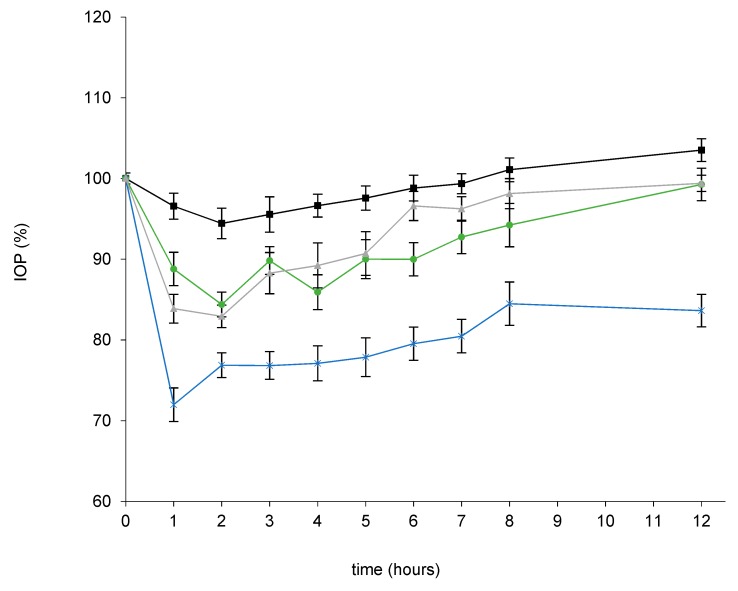
IOP % profile of the following formulations: (▲) Marketed formulation (TM 0.5%), (●) TMGNP3 (0.1%), (**×**) TMGNP3 (0.1%) + HPMC 0.3% and (■) blank gelatin nanoparticles used as control.

In a second set of experiments, the nanoparticle formulation that produced a similar hypotensive effect to the commercial preparation was combined with HPMC: TM-GNP3 (TM 0.1%) + HPMC 0.3%. This hybrid system promoted an increment in drug efficacy for all parameters evaluated: Maximal % IOP reduction (1.5-fold higher than the marketed preparation), Maximal mmHg reduction (1.5-fold higher than the commercial reference), and drug bioavailability expressed as AUC _(0–12)_ (2.5-fold higher than the marketed formulation) these values were statistically significantly higher (*p* values at Table 5) (Figure 5 and Table 6).

Furthermore, the hypotensive effect reached a maximum value 1 h after the instillation of TM-GNP3+HPMC and, interestingly, it was maintained for 12 h, instead of 8 h, as observed with the commercial preparation and TM-GNP formulations that did not contain HPMC.

## 4. Discussion

The need for novel drug delivery systems is a major challenge in obtaining safe and effective therapies for managing IOP in glaucoma. However, the ocular surface barriers entail a challenge for drug penetration. To overcome this limitation high drug concentration and frequent instillations are needed, which can lead to important systemic side effects and low patient compliance.

The potential of nanoparticles to decrease the adverse effects associated with beta-blockers drugs in glaucoma have been widely remarked [50,66,67]. The present work tries to offer a biodegradable, well tolerated and effective nanoparticulate system to address TM for IOP reduction. With this idea, gelatin was selected as the nanocarrier component because of its well-known biocompatibility [68]. Gelatin can present different bloom index (measured by gel strength) that indicates the amount of triple-helix content, determining mechanical, rheological and thermal properties, as well as gelatin biodegradation that regulates drug release [66,67] Among the different available gelatines, in this work a high Bloom gelatin (225 g bloom values; 50,000–100,000 g/mol) was chosen. This characteristic leads to a raised melting point and enhanced stability [69] and also to a short gelling time heading to rapid nanoparticle formation [69,70]. Other authors have also employed GNPs for TM delivery purposes. In some cases, glutaraldehyde has been used as a crosslinked agent [71]. However, in this work, we have employed a less cytotoxic crosslinker to create the gelatin nanogels, glyoxal [59], and an extensive dialysis final step in the nanoparticles manufacturing to guarantee the elimination of any unreacted and undesired compound. Low particle sizes such as those obtained in the present work (193.2 ± 20.7 nm) avoid irritation or discomfort over the eye surface [72,73,74], and also it has been established that this size range can improve drug permeation within the corneal layer [47]. The result of the encapsulation efficiency of TM (58.7 ± 3.82%) can be explained since the near-neutral potential of the particles increases affinity between timolol and the nanoparticle former material gelatin [75]. All these characteristics make the GNP nanoparticles developed in this work very suitable for ocular topical administration in eye drops.

The formulation of gelatin nanoparticles containing the same TM dose as the marketed formulation prepared in this work was able to control *in vitro* the release of timolol. The release profile was characterized by a fast initial release during the first 24 h (80.50; 79.22; 77.00% for PBS, Protease and MMP-2 media respectively), probably due to the rapid delivery of the drug adsorbed onto the nanoparticle surface [51]. The posterior particles gelling would hinder the drug diffusion from its inner core to the release media leading to a low release fashion extended for three additional days [76,77]. *In vitro* release studies were also performed in media containing protease or MMP-2 at the equivalent concentrations observed on the ocular surface in glaucomatous patients suffering inflammation events [53,54,55]. According to our data, these nanoparticles were resistant to these media, being able to protect the active compound and to progressively release it even at these conditions, probably due to the high density of the crosslinked gelatin matrix [78].

Although, an important number of different nanodevices composed by diverse materials are being evaluated for topical ocular drug delivery purposes in the past decades, not all of them have been properly tested in terms of ocular tolerance, which is an imperative previous step in order to guarantee good *in vivo* tolerance of the proposed systems [22]. That is why, in this work, we have *in vitro* corroborated the selection of nanoparticle concentrations with optimal ocular tolerance. For this purpose, *in vitro* tolerance studies were performed in order to establish nanoparticles concentrations suitable for *in vivo* efficacy studies. *In vitro* tolerance studies showed a concentration-dependent reduction of cell viability for nanoparticles loaded with timolol maleate. Considering that non-loaded nanoparticles and gelatin solution resulted well tolerated even at the highest concentration studied (30 mg/mL) and that both the timolol maleate solution and the marketed formulation (0.5% TM) also showed cell viability reduction after 24h of contact, it seems rational to blame the drug and not the polymer for this phenomena. In fact, other authors have already demonstrated that high concentrations of this drug produce a cytotoxic effect in Chang’s conjunctival cell line [79]. The *in vivo* acute tolerance test performed confirmed that a high concentration (30 mg/mL) of non-loaded gelatin nanoparticles were well tolerated, supporting the suitability of the drug delivery system proposed in this work. Unfortunately, the exposure of animals’ eyes to very frequent doses of timolol (an instillation each 30 min) was not ethically possible, so this *in vivo* acute tolerance study could not be performed with loaded nanoparticles. At this point it was reasonable to limit the use of timolol maleate loaded nanoparticles at concentrations lower than 20 mg/mL (well tolerated in cell cultures) for *in vivo* efficacy studies. In fact, in order to mimic the clinical treatments currently performed with the commercial preparation (0.5% TM) the highest concentration of TM-GNP (*in vivo* tested) was fixed at 16 mg/mL (0.5% TM) for efficacy studies. Additionally, 8 mg/mL and 3 mg/mL TM-loaded nanoparticles concentrations were also tested. According to our results, the inclusion of timolol maleate in gelatin nanoparticles promoted a higher ocular bioavailability in comparison to its administration in the marketed formulation (1.7-fold increments of TM-GNP16 versus the commercial preparation). In fact, the administration of only 0.1% of timolol maleate included in nanoparticles (3 mg of nanoparticles/mL) offered the same hypotensive effects in terms of maximal % IOP reduction and maximal mmHg reduction and also of drug bioavailability (expressed as AUC _(0–12)_) than the marketed preparation. The employment of 5-fold less TM to reach the same hypotensive effect might be very interesting in clinic. Accordingly, several studies have demonstrated that ophthalmic treatments with TM 0.1% achieve considerably lower plasma concentrations compared to TM 0.5% [80,81,82] and thus, reducing the systemic effects after administration of the commercial formulation. In the present study the commercial formulation (TM 0.5%) was only able to reach a 21% IOP reduction in normotensive rabbits, meanwhile, TM-GNP8 (TM 0.25%) was able to achieve a 26% decrease including half of the dose and TM-GNP16 (TM 0.5%) promoted IOP decrease over 30%. Significant differences have been observed in all the cases (*p* < 0.05). The aforementioned results could be explained by the mucoadhesive properties of gelatin, leading to a higher retention time on the ocular surface [48] and higher corneal penetration of the nanoparticulate system [47,51,83]. These data demonstrate that timolol loaded gelatin nanoparticles can result in a very promising strategy for ocular delivery of hypotensive compounds and that, interestingly, target pressure can be modulated employing different concentrations of TM-GNP. In all these efficacy studies, the hypotensive effect was observed for 8 h after a single administration. The combination of TM-GNP3 with a viscous solution composed by HPMC 0.3% statistically improved (*p* < 0.05) not only the IOP reduction but also the reaching values of 30% and the maximal mmHg reduction, as well as faster maximal action (t_max_ = 1 h). Furthermore, the hypotensive effect was extended for four additional hours reaching a total of 12 h of pharmacological activity after a single instillation and a raised ocular bioavailability (2.5-fold higher than the one observed for the marketed formulation), due to the beneficial combination of nanosystems and the viscous agent [24]. The incorporation of HPMC has the effect of not only enhancing efficacy over TM-GNPs, but also it has been widely demonstrated that the incorporation of HPMC in antiglaucomatous eye drops increases patient compliance. This fact has been explained due to the recession of diverse side effects associated with chronic eye drops administrations such as DED [84,85].

## 5. Conclusions

Gelatin nanoparticles alone or in combination with viscous polymeric solutions have been demonstrated as a promising alternative to deliver beta-blockers for controlling IOP in glaucoma, both in the presence or absence of damaged ocular surface and can be an interesting tool for personalized treatments. The platform created by the combination of gelatin nanoparticles and well tolerated viscous agents can not only lead to higher patient compliance and medical adherence, but also can offer additional benefits in terms of tolerance for chronic treatments.

## Figures and Tables

**Table 1:** Grading system for *in vivo* tolerance evaluation signs.

Grade	Discomfort	Cornea	Conjunctiva	Discharge	Lids
0	No reaction	No alterations	No alterations	No discharge	No swelling
1	Blinking	Mild Opacity	Mild hyperemia/mild oedema	Mild discharge without moistened hair	Mild swelling
2	Enhanced blinking/intense tearing/vocalizations	Intense opacity	Intense hyperemia/intense oedema/haemorrhage	Intense discharge with moistened hair	Obvious swelling

**Table 2:** Initial burst and release at 24 h TM-NPs in the different release media employed (PBS, PBS with protease and PBS with MMP-2).

Release Media	Initial Burst (0.5 h)	24 h
PBS	5.90 ± 1.04	80.46 ± 1.44%
PBS and Protease (60 ng/mL)	4.88 ± 1.29%	80.46 ± 9.17%
PBS and MMP-2 (60 ng/mL)	4.50 ± 0.83%	77.00 ± 11.87%

**Table 3:** Maximal % IOP reduction, Maximal mmHg reduction, t_max_, hypotensive effect duration (hours) and the area under the curve AUC (0–12h) from the developed formulations.

Formulation	Maximal % IOP Reduction	Maximal mmHg Reduction	t_max_	Hypotensive Effect Duration (hours)	AUC (0–12)
Marketed formulation (TM 0.5%)	21.11 ± 1.72	3.23 ± 0.26	1.5	8	83.77 ± 11.71
TM-GNP16 (TM 0.5%)	30.09 ± 1.85	4.33 ± 0.30	2.0	8	143.63 ± 14.16
TM-GNP8 (TM 0.25%)	26.39 ± 1.38	3.75 ± 0.23	2.0	8	124.37 ± 11.89
TM-GNP3 (TM 0.1%)	20.46 ± 1.41	2.98 ± 0.20	2.0	8	102.44 ± 13.47

**Table 4:** *p*-values obtained after one-way ANOVA comparison between the reference commercial formulation and timolol-loaded nanoparticle formulations. (* significant differences *p* < 0.05).

	AUC _(0–12)_	Maximal mmHg Reduction %	Maximal mmHg Reduction
**TM-GNP3 (TM 0.1%)**	*p* = 0.1581	*p* = 0.9376	*p* = 0.7339
**TM-GNP8 (TM 0.25%)**	*p* = 0.0075 *	*p* = 0.0119 *	*p* = 0.0279 *
**TM-GNP16 (TM 0.5%)**	*p* = 0.0009 *	*p* = 0.0011 *	*p* = 0.0011 *

**Table 5:** *p*-values obtained after one-way ANOVA comparison between the reference marketed formulation and TM-GNP3 with and without viscous HPMC. (* significant differences *p* < 0.001).

	AUC _(0–12)_	Maximal mmHg Reduction %	Maximal mmHg Reduction
**TM-GNP3 (TM 0.1%)**	*p* = 0.1581	*p* = 0.9376	*p* = 0.7339
**TM-GNP3 (TM 0.1%) + HPMC 0.3%**	*p* < 0.0001 *	*p* = 0.0007 *	*p* = 0.0010 *

**Table 6:** Maximal % IOP reduction ± SEM; t_max_ acquired by the median of first maximum effect and AUC is the difference of IOP (%) time curve from 0–12 h ± SE.

Formulation	Maximal % IOP Reduction	Maximal mmHg Reduction	t_max_	Hypotensive Effect Duration (hours)	AUC (0–12)
Marketed formulation (TM 0.5%)	21.11 ± 1.72	3.23 ± 0.26	1.5	8	83.77 ± 11.71
TM-GNP3 (TM 0.1%)	20.46 ± 1.41	2.98 ± 0.20	2.0	8	102.44 ± 13.47
TM-GNP3 (TM 0.1%) + HPMC 0.3%	29.64 ± 1.60	4.65 ± 0.24	1.0	12	213.85 ± 13.75
